# Clinical and CT image features for survival prediction in severe pneumonia during the SARS-CoV-2 Omicron wave

**DOI:** 10.3389/fmed.2025.1663710

**Published:** 2026-01-09

**Authors:** Wei Xu, Jing Zhao, Teng Wang, Jingjiang Lai, Jingliang Wang, Fengxian Jiang, Cuiyan Wang, Guobin Fu

**Affiliations:** Shandong Provincial Hospital affiliated to Shandong First Medical University, Jinan, China

**Keywords:** severe acute respiratory syndrome coronavirus 2, severe COVID-19 pneumonia, CT imaging features, clinical features, survival prediction

## Abstract

**Background:**

Identifying prognostic factors for severe COVID-19 pneumonia during the Omicron wave remains crucial for early risk stratification and improving patient outcomes. This study aimed to identify and analyze key clinical and CT imaging features associated with survival in patients with severe pneumonia caused by the SARS-CoV-2 Omicron variant.

**Methods:**

This retrospective study included patients presenting to the emergency department of Shandong Provincial Hospital (December 2022–January 2023) with confirmed SARS-CoV-2 Omicron infection and severe pneumonia. Clinical/laboratory data and CT imaging features were systematically collected and evaluated. Patients were randomly divided into training (70%) and validation (30%) cohorts. Univariate and multivariate analyses were rigorously applied to identify significant baseline clinical and CT imaging features associated with survival. A predictive nomogram was constructed based on the selected feature combination.

**Results:**

Among 1,739 COVID-19 patients, 151 (8.68%) had severe pneumonia (median age 75, 70.1% male). Multivariate logistic regression analysis identified a critical combination of features independently associated with survival: CT findings of pleural effusion (*p* = 0.008) and cardiac enlargement (*p* = 0.008), along with clinical/laboratory factors including reduced baseline pulse oxygen saturation (*p* = 0.034), elevated SAA (*p* = 0.020), elevated GLU (*p* = 0.022), and reduced Ca concentration (*p* = 0.029). The nomogram integrating these combined features demonstrated good predictive performance for in-hospital mortality (AUC: training cohort 0.914, validation cohort 0.802).

**Conclusion:**

This study identifies a distinct combination of clinical and CT imaging features (pleural effusion, cardiac enlargement, low SpO2, elevated SAA, elevated GLU, low Ca) as key independent prognostic factors for survival in severe Omicron pneumonia. The predictive tool based on this feature combination shows significant clinical utility. These preliminary findings provide critical insights for early risk assessment and targeted management, facilitating improved patient prognosis.

## Introduction

The coronavirus disease 2019 (COVID-19) pandemic, caused by severe acute respiratory syndrome coronavirus 2 (SARS-CoV-2), has been one of the greatest threats to public health in the 21st century with >400 million identified cases and >5.9 million deaths reported worldwide (as of 29 March 2023) (1, 2). SARS-CoV-2 infection may remain asymptomatic in the early stages, until the emergence of severe pneumonia, dyspnea, organ dysfunction, and even death (3, 4). Although survival rates have gradually improved by the development of successful treatment protocols for moderate and severe patients, the disease continues to claim lives and containment is proven difficult (2, 5).

Overall ICU mortality has ranged from 30% to 50% throughout the pandemic and is influenced by many factors including ICU strain and location (epicenter vs. non-epicenter) (4, 6–8). Severe disease is associated with advanced age, male sex, residence in a nursing home, underlying comorbidities (e.g., cardiovascular disease, diabetes, chronic lung disease, hypertension etc.) and higher computed tomography (CT) severity score (4, 9). Chest X-rays or CT exams are now the primary imaging modality for clinical management. The Fleischner Society released a consensus statement discussing the use of imaging in patient assessment, diagnosis, and risk stratification, noting that in COVID-19 positive patients, imaging can determine baseline lung status and identify potential cardiopulmonary abnormalities that may contribute to risk stratification for clinical deterioration (10). Previous studies have predicted outcomes in young and middle-aged adults with COVID-19 based on initial chest radiographs with lung area severity scores (11). However, CT is usually the first-line test for COVID-19 in China because it is more sensitive in detecting early lung lesions (12).

The conclusions of previous studies are based on clinical data from the original strain or Delta variant, but there are questions about the applicability of the currently emerging Omicron variant. The Omicron variant represents the most diverse strain of SARS-CoV-2, raising global concerns due to its significant transmissibility and capacity to evade immune responses (13). Because of its heightened transmissibility, Omicron has quickly supplanted Delta as the predominant variant in numerous areas, becoming the primary strain in the subsequent widespread outbreak of COVID-19 pneumonia following the relaxation of outbreak policies in China in December 2022. Data on surveillance provided by the Chinese Center for Disease Control and Prevention indicate that between 1 December 2022, and 23 January 2023, a total of 10,165 valid genome sequences of SARS-CoV-2 from domestic cases were documented across the country, all identified as Omicron variants, encompassing 24 distinct lineages. The predominant strains responsible for the epidemic are BA.5.2, accounting for 70.2%, and BF.7, representing 28.3% (14). The purpose of this study was to document the clinical and CT imaging characteristics of patients with severe pneumonia infected with the Omicron variant and to identify factors that affect patient prognosis. In addition, we used the collected data to develop a mortality risk prediction model in order to assess patients’ disease at the beginning of their visit with infected COVID-19 patients, to facilitate the clinical application of COVID-19 management, and to improve patient prognosis.

## Materials and methods

### Study design and populations

The retrospective study enrolled patients with COVID-19 pneumonia who first attended Shandong Provincial Hospital affiliated to Shandong First Medical University, between 2022.12.01 and 2023.01.10. Based on the World Health Organization (WHO) Interim Guidelines for the Treatment of Novel Coronavirus Pneumonia (10th edition) published by the National Health Commission of China, COVID-19 pneumonia is diagnosed. Patients meeting the following inclusion criteria were included in this study: (1). Positive SARS-COV-2 RT-PCR by pharynx swab specimen; (2). Typical chest CT presentation of combined COVID-19 pneumonia. Exclusion criteria: (1). patients with unconfirmed COVID-19 pneumonia; (2). incomplete baseline clinical information; and (3). patients with no clinical outcome (discharge/death) during the study period. Severe pneumonia caused by COVID-19 is characterized by the fulfillment of at least one of the following criteria: (1). a respiratory rate of 30 breaths per minute or higher accompanied by shortness of breath; (2). an oxygen saturation level of 93% or lower while at rest without supplemental oxygen; (3). an oxygenation index (the ratio of arterial partial pressure of oxygen to oxygen concentration) of 300 mmHg or less (with 1 mmHg equivalent to 0.133 kPa) or an arterial partial pressure of oxygen equal to or below 60 mmHg on room air at rest; (4). a noticeable deterioration in clinical symptoms along with lung imaging that reveals a significant increase of over 50% in the affected area within a time frame of 24 to 48 h.

**FIGURE 1:**
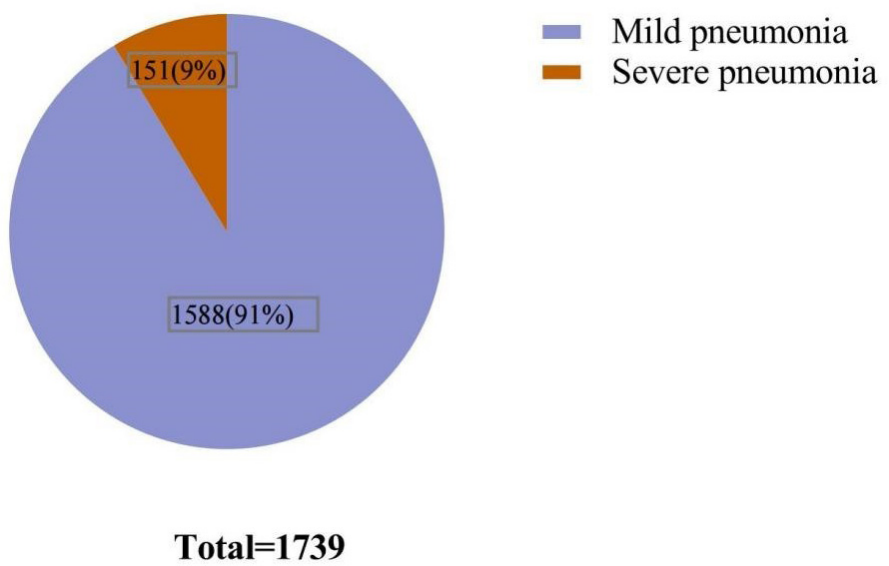
Proportion of patients with severe COVID-19 pneumonia among all patients with COVID-19 pneumonia.

**FIGURE 2:**
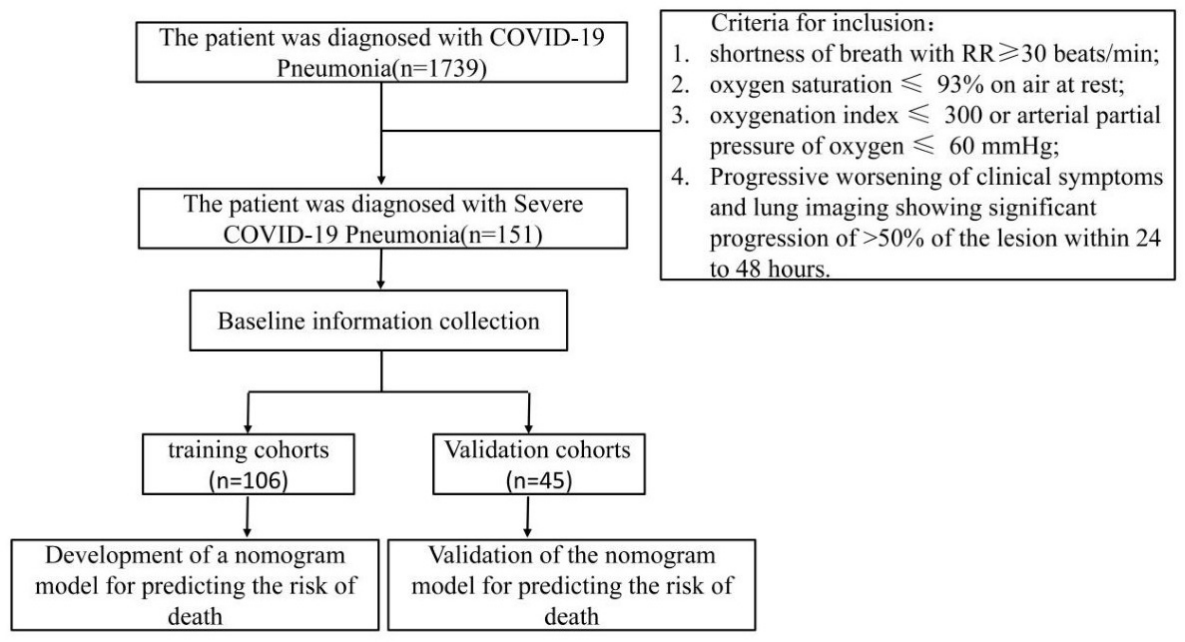
Flow chart of cases selection.

A total of 1,739 patients diagnosed with COVID-19 pneumonia participated in this study, among whom there were 151 patients experiencing severe pneumonia (Figure 1). In Figure 2, the selection process of the study population comprising 151 patients diagnosed with severe COVID-19 pneumonia is illustrated. The patients were systematically randomized into two distinct cohorts: the training cohort and the validation cohort. This division was executed in a ratio of 7:3, ensuring that the majority of participants were allocated to the training group while a smaller subset was designated for validation purposes. This methodological approach facilitates a comprehensive analysis and validation of the findings derived from the research. The ethics committee of Shandong Provincial Hospital affiliated with Shandong First Medical University approved the investigation (ethics number: SWYX: No. 2022-593). The study adhered to the Statement of Standards for Reporting Diagnostic Accuracy Studies, and the hospital’s ethics committee waived the requirement for written informed consent from patients with COVID-19 pneumonia (15).

### Clinical and laboratory data collection

All patients were admitted to the Shandong Provincial Hospital affiliated with the Shandong First Medical University, and their pharyngeal swab specimens were tested for SARS-CoV-2; patients who tested positive by real-time RT-PCR were considered confirmed cases.

**FIGURE 3:**
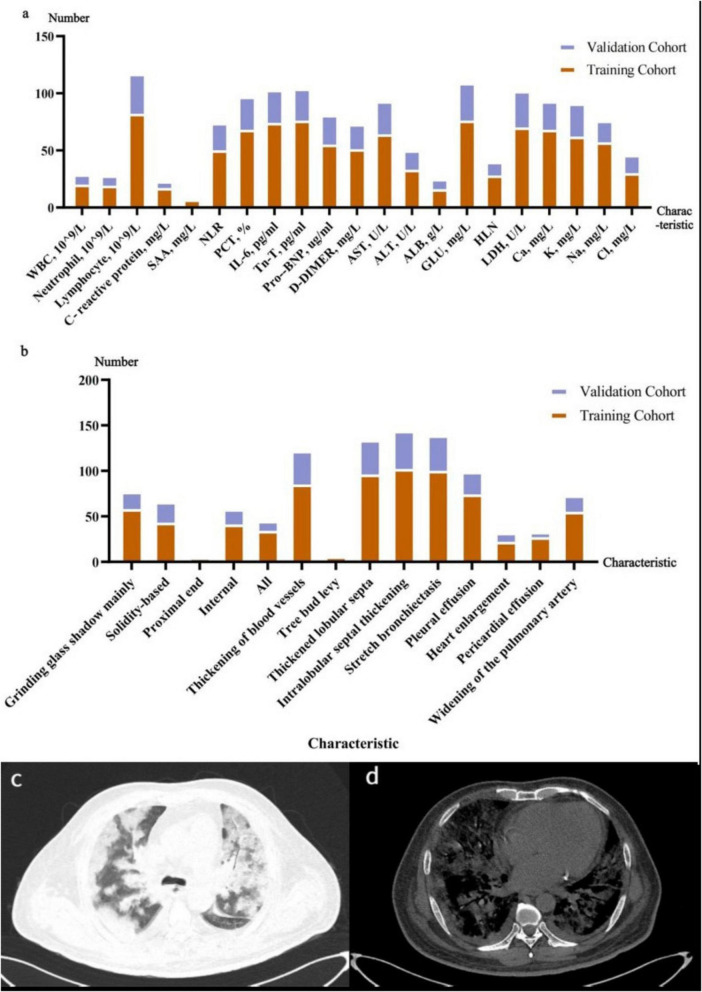
**(a)** Baseline laboratory characteristics of patients with severe COVID-19 on admission. **(b)** Baseline imaging characteristics of patients with severe COVID-19 on admission. **(c,d)** Male, 66 years old, with intermittent fever for 12 days and chest tightness and shortness of breath for 2 days. COVID-19 nucleic acid antigen detection (+) **(c)** Lung window with multiple nodular, patchy hyperdense shadows in both lungs, locally dense and poorly defined, with air bronchial signs visible within; **(d)** Mediastinal window, image shows multiple solid dense shadows in both lungs, enlarged heart and bilateral pleural effusions.

After thorough consultation with respiratory and imaging experts and an extensive review of the literature related to COVID-19 prognosis, we finally identified a set of clinical, laboratory and imaging baseline characteristics for inclusion in the study. A number of clinical trials have now confirmed that various factors, such as Hypertension (*P* = 0.038), higher neutrophil-to-lymphocyte ratio (NLR) (*P* = 0.001), increased NT-proBNP (*P* < 0.001), age ≥ 70 years (OR = 1.184, 95% CI 1.061–1.321), panting (breathing rate ≥ 30/min) (OR = 3.300, 95% CI 2.509–6.286), lymphocyte count < 1.0 × 109/L (OR = 2.283, 95% CI 1.779–3.267), and interleukin-6 (IL-6) > 10 pg/ml (OR = 3.029, 95% CI 1.567–7.116) are significantly associated with the poor prognosis of patients with COVID-19 (16, 17). Therefore, in this study, we collated and included laboratory and imaging indicators that had been mentioned in the literature and were clinically available. These included: routine blood (WBC, Neutrophil and Lymphocyte), inflammatory factors (C-reactive protein, SAA, PCT, IL-6 and NLR), cardiac enzyme profiles (Tn-T and Pro–BNP), coagulation (D-DIMER), liver and kidney function (AST, ALT, LDH, ALB, HLN, and GLU), blood biochemistry (Ca, K, Na, Cl), and imaging features (Nature of lesion, Bronchial wall thickening, Thickening of blood vessels, Tree bud levy, Thickened lobular septa, Intralobular septal thickening, Stretch bronchiectasis, Pleural effusion, Heart enlargement, Pericardial effusion and Widening of the pulmonary artery) (Figure 3). Patients with incomplete baseline clinical information were excluded, as stated in the exclusion criteria. Among the baseline clinical characteristics were basic patient information, major symptoms and comorbidities, all of which were obtained at the time of the patient’s first admission (Table 1). Baseline laboratory characteristics included: routine blood (WBC, neutrophils, lymphocytes), inflammatory factors (c-reactive protein, SAA, PCT, IL-6, NLR), cardiac enzyme profile (Tn-T, Pro- BNP), coagulation (D-DIMER), liver and kidney function (AST, ALT, LDH, ALB, HLN, GLU), blood biochemistry (Ca, K, Na, Cl); the Cut-off value of each index in predicting the prognosis of patients with COVID-19 pneumonia was used as the basis for grouping. Cut-off values for continuous variables were determined based on clinical standards and previous studies. For example, SpO2 ≤ 93% is a criterion for severe COVID-19 according to WHO guidelines, and GLU > 7.5 mmol/L is a common threshold for hyperglycemia. For imaging features like heart enlargement, it was defined as a cardiothoracic ratio > 0.5 on axial CT images, as assessed by experienced radiologists.

**TABLE 1:** Demographic and clinical features of patients in the training and validation cohorts.

Characteristic no. (%)	All patients (*n* = 151)	Training cohort (*n* = 106)	Validation cohort (*n* = 45)
Age, [median (IQR)]	75 (36–96)	–	–
Male	106 (70.1%)	82 (77.3%)	24 (53.3%)
Vaccination	72 (47.6%)	52 (49.0%)	20 (44.4%)
**Symptoms**			
Cough	69 (45.6%)	46 (43.3%)	23 (51.1%)
Fever	104 (68.8%)	74 (69.8%)	30 (66.6%)
Breath shortness	92 (60.9%)	70 (66.0%)	22 (48.8%)
**Comorbidities**			
Hypertension	79 (52.3%)	50 (47.1%)	29 (64.4%)
Diabetes	46 (30.4%)	30 (28.3%)	16 (35.5)
Cardiovascular disease	65 (43.0%)	43 (40.5%)	22 (48.8%)
Chronic lung disease	19 (12.5%)	16 (15.0%)	3 (6.6%)
Chronic liver disease	5 (3.3%)	4 (3.7%)	1 (2.2%)
Chronic kidney disease	15 (9.9%)	12 (11.3%)	3 (6.6%)
Cancer	18 (11.9%)	16 (15.0%)	2 (4.4%)
Autoimmune system disease	8 (5.2%)	5 (4.7%)	3 (6.6%)
**Basic vital signs (>cut-off)**			
Temperature °C	32 (21.1%)	23 (21.6%)	9 (20.0%)
Heart rate	59 (39.0%)	41 (38.6%)	18 (40.0%)
Pulse Oximetry	69 (45.6%)	41 (44.5%)	28 (47.4%)
**Laboratory findings to admission (>cut-off)**			
WBC	29 (19.2%)	18 (19.5%)	11 (18.6%)
Neutrophil	28 (18.5%)	18 (19.5%)	10 (16.9%)
Lymphocyte	117 (77.4%)	70 (76.0%)	47 (79.6%)
C-reactive protein	23 (15.2%)	11 (11.9%)	12 (20.3%)
SAA	9 (5.9%)	3 (3.2%)	6 (10.1%)
NLR	46 (30.4%)	24 (26%)	22 (37.2%)
Tn-T	104 (68.8%)	64 (69.5%)	40 (67.7%)
Pro–BNP	81 (53.6%)	47 (51%)	34 (57.6%)
D-DIMER	73 (48.3%)	45 (48.9%)	28 (47.4%)
AST	93 (61.3%)	61 (66.3%)	32 (54.2%)
ALT	50 (33.1%)	31 (33.6%)	19 (32.2%)
ALB	25 (16.5%)	16 (17.3%)	9 (15.2%)
GLU	109 (72.1%)	66 (71.7%)	43 (72.8%)
HLN	40 (26.4%)	25 (27.1%)	15 (25.4%)
LDH	101 (66.8%)	60 (65.2%)	41 (69.4%)
PCT	97 (64.2%)	58 (63.0%)	39 (66.1%)
IL-6	103 (68.2%)	62 (67.3%)	41 (69.4%)
**Imaging characteristics**			
Nature of lesion	–	–	–
Grinding glass shadow mainly	77 (50.9%)	49 (53.2%)	28 (47.4%)
Solidity-based	66 (43.7%)	40 (43.4%)	26 (44.0%)
**Bronchial wall thickening**			
Proximal end	6 (3.9%)	4 (4.3%)	2 (3.3%)
Internal	58 (38.4%)	37 (40.2%)	21 (35.5%)
All	45 (29.8%)	30 (32.6%)	15 (25.4%)
Thickening of blood vessels	122 (80.7%)	73 (79.3%)	49 (83.0%)
Tree bud levy	8 (5.2%)	5 (5.4%)	3 (5.0%)
Thickened lobular septa	134 (88.7%)	81 (88.0%)	53 (89.8%)
Intralobular septal thickening	144 (95.3%)	89 (96.7%)	55 (93.2%)
Stretch bronchiectasis	139 (92.0%)	88 (95.6%)	51 (86.4%)
Pleural effusion	105 (69.5%)	65 (70.6%)	40 (67.7%)
Heart enlargement	31 (20.5%)	16 (17.3%)	15 (25.4%)
Pericardial effusion	33 (21.8%)	15 (16.3%)	18 (30.5%)
Widening of the pulmonary artery	73 (48.3)	41 (44.5)	32 (54.2%)

### Image analysis

Computed tomography scans for each patient collected during their hospital stay were sourced from the cloud-based data storage system. The original chest radiograph and CT scan were characterized as those taken upon admission or within one week of the onset of symptoms. Follow-up chest radiographs were performed every 2–3 days until the patient was discharged (18).

All the patients’ CT features were independently assessed on all data sets by two radiologists with 10 years of experience in reading chest CT images, who had no knowledge of the clinical and laboratory results. The analysis focused on the extent and patterns of pneumonia observed in both initial and follow-up CT scans. The severity of pneumonia across all lung zones on the CT scans was rated on a scale from 0 to 2, where a score of 0 indicates no pneumonia, a score of 1 indicates 1%–25% involvement, and a score of 2 indicates more than 25% involvement (18, 19). Pneumonia patterns identified in the CT images were classified as typical, indeterminate, atypical, or negative, according to the RSNA Expert Consensus Statement (18, 20). A typical appearance was characterized by peripheral bilateral ground-glass opacities (GGOs) or multifocal round GGOs, either with or without consolidation, and possibly featuring intralobular lines or a reverse halo sign. An indeterminate appearance referred to the presence of GGOs, with or without consolidation, but lacking definitive typical characteristics. In contrast, an atypical appearance was noted when typical or indeterminate features were absent, while lobar and/or segmental consolidation was observed without the presence of GGOs, distinct centrilobular nodules, lung cavitation, or smooth interlobular septal thickening accompanied by pleural effusion (18). We have also found some other interesting imaging features, including bronchial wall thickening, thickening of blood vessels, tree bud levy, stretch bronchiectasis, heart enlargement, pericardial effusion and widening of the pulmonary artery.

### Model construction

After recruitment, all patients were randomly grouped into a training cohort and a validation cohort in a 7:3 ratio. A nomogram predicting survival in patients with severe COVID-19 pneumonia was constructed in the training cohort based on baseline characteristics, and then validated in a separate validation cohort.

### Statistical analysis

Categorical variables are described using frequencies and proportions, and continuous variables are described using medians and quartiles. Univariate logistic regression analysis was used to identify clinically relevant variables associated with death in patients with severe COVID-19 pneumonia in the training cohort, variables showing univariate relationships associated with death in patients with severe COVID-19 pneumonia (*p* < 0.05) were entered into a multivariate logistic regression analysis, and improvements in goodness of fit were assessed by reducing the Akaike information criterion for backward stepwise selection. If the number of events was too small to calculate an OR, the variables were excluded; a final model was created by selecting baseline clinical indicators from the training cohort; and a nomogram was constructed from the overall data of the training cohort based on the selected final model. To assess the ability of the nomogram model to differentiate the risk associated with death in patients with severe COVID-19 pneumonia. The area under the subject’s working curve (AUC) and 95% CI were calculated and compared to the AUC curve and 95% CI for each independent variable of interest in the training cohort. To assess the agreement between nomogram predictions and actual observations in the training cohort, 1,000 resamples (with replacement) were set up and calibration curves were created. To assess the clinical applicability of the predictive nomogram, a DCA curve analysis was performed by quantifying the net benefit at different threshold probabilities of death in patients with severe COVID-19 pneumonia. The net benefit was defined as the proportion of true positives minus the proportion of false positives, as measured by the relative risk of false positive and false negative outcomes. To assess the internal validity of the model, the model was applied to an independent dataset from Shandong Provincial Hospital affiliated to Shandong First Medical University. The internal validity of the model was assessed using AUC, calibration and DCA curve analysis for the risk associated with death in patients with severe COVID-19 pneumonia, respectively. All statistical analyses were performed using R (version 4.2.1) and *p* < 0.05 was considered statistically significant.

## Results

### Clinical characteristics of patients with COVID-19

A total of 1,739 confirmed cases of COVID-19 with viral pneumonia first attended at Shandong Provincial Hospital affiliated to Shandong First Medical University between 2022-12-01 and 2023-01-10 were enrolled in this study. A total of 151 patients were diagnosed with severe COVID-19 after screening according to stringent entry criteria, the rate of serious illness was 8.68% (Figure 1). Table 1 display the fundamental characteristics of seriously ill patients. In this study, the median age of the patients was 75 years (range: 36–96 years), and 106 (70.1%) were males. A total of 72 (47.6%) of these patients had previously received at least one dose of the COVID-19 vaccine. The majority of patients in the entire cohort experienced fever (68.8%) and chest congestion (60.9%), whereas 69 patients (45.6%) had a cough. The most prevalent comorbidity was hypertension (52.3%), followed by cardiovascular disease (43%). Diabetes, chronic pulmonary disease, and cancer accounted for 30.4%, 12.5%, and 11.1% of all patients, respectively. A total of 106 patients (70%) were allocated to the training cohort, while 45 patients (30%) were assigned to the validation cohort. All patient information was provided, and the majority of characteristics did not differ substantially between the two groups (Table 1).

### Laboratory and imaging characteristics of patients with COVID-19

The Cut-off values of each index in the prognosis prediction of patients with COVID-19 were used as the basis for their grouping, respectively (Table 2).

**TABLE 2:** The calculations, cut-off points of enzymes and blood cytology indicators.

Indicators	Cut-off
**Basic vital signs**	
Temperature, °C	37.0
Heart rate	84
Breathing rate	20
Pulse Oximetry	93
**Laboratory findings to admission (>cut-off)**	
**Routine blood**	
WBC, 10^9/L	12.05
Neutrophil, 10^9/L	10.63
Lymphocyte, 10^9/L	0.40
**Inflammatory factors**	
C- reactive protein, mg/L	167.93
SAA, mg/L	1134.5
NLR	10.71
PCT, %	0.075
IL-6, pg/ml	6.955
**Cardiac enzyme profiles**	
Tn-T, pg/ml	12.9
Pro–BNP, μg/ml	525
D-DIMER, mg/L	1.77
**Liver and kidney function**	
AST, U/L	28.5
ALT, U/L	36.5
ALB, g/L	38.05
GLU, mg/L	7.5
HLN	112.63
LDH, U/L	312.93
**Blood biochemistry**	
Ca, mg/L	2.05
K, mg/L	3.84
Na, mg/L	133.95
Cl, mg/L	103.07

WBC, white blood cell; LYMPH, lymphocyte; SAA, Serum amyloid A; NLR, neutrophil-to-lymphocyte ratio; PCT, procalcitonin; IL-6, interleukin- 6; Tn-T, troponin T; AST, aspartate aminotransferase; ALT, alanine aminotransferase; ALB, albumin; GLU, glutamic acid; LDH, lactate dehydrogenase.

### Treatment and outcome

Oxygen therapy was the most common treatment for patients hospitalized with severe COVID-19. All patients hospitalized with severe COVID-19 received oxygen therapy, which was mainly nasal cannula oxygen (83.4%), mask oxygen (35.0%) and mechanical ventilation (13.2%); followed by antibiotic therapy (96.6%) and glucocorticoid therapy (90.7%). A total of 78.1%, 20.5%, and 13.2% of patients received antiviral drugs, intravenous immunoglobulin and Chinese medicine, Antiviral drugs, intravenous immunoglobulin and herbal medicines were used in 78.1%, 20.5%, and 13.2% of patients. Antivirals mainly consisted of azelvadine tablets and nematovir/ritonavir tablet combination packs (Table 3).

**TABLE 3:** Treatment and outcome of patients in the training and validation cohorts.

Characteristic no. (%)	All patients (*n* = 151)	Training cohort (*n* = 106)	Validation cohort (*n* = 45)
**Treatment and outcome**			
Oxygen inhalation	151 (100%)	106 (100%)	45 (100%)
Antibacterial therapy	146 (96.6%)	102 (96.2%)	44 (97.7%)
Glucocorticoids	137 (90.7%)	96 (90.5%)	41 (91.1%)
Antivirus therapy	118 (78.1%)	80 (75.4%)	38 (84.4%)
Intravenous immunoglobulin therapy	31 (20.5%)	23 (21.6%)	8 (17.7%)
Herbal medicines	20 (13.2%)	14 (13.2%)	6 (13.3%)
**Outcome**			
Discharged	92 (61%)	63 (59.5%)	29 (64.4%)
Died	59 (39%)	43 (40.5%)	16 (35.5%)

**FIGURE 4:**
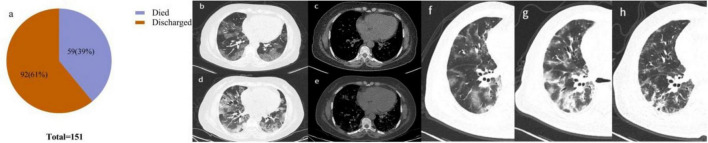
**(a)** Disease regression after treatment of patients with severe COVID-19 pneumonia. **(b–e)** Female, 69 years old, with fever, cough and chest tightness for 6 days. **(b,c)** 2022-12-29 CT, multiple nodular, lattice-like and patchy hyperdense shadows in both lungs with slight local pleural thickening; **(d,e)** On 2023-01-03 CT, the lesion was more extensive than before, with increased density, localized densities and localized thickening of the pleura bilaterally. **(f–h)** Male, 67 years old, fever with cough for 10 days. **(f)** 2022-12-25 CT, multiple patchy, banded ground glass density shadows are seen in the lung field with a predominant subpleural and peribronchial vascular bundle distribution with blurred, partially dense margins. **(g)** 2023-01-01 CT, most lesions are more extensive and denser than before, with overall progression from before. **(h)** 2023-01-09 CT, partially less extensive and less dense than before, overall better than before.

Among patients with severe COVID-19, 92 (61%) were discharged after cure, with a mean hospital stay of 21 days; 59 (39%) died during their hospital stay, with a mean hospital stay of 19 days (Figure 4).

### Independent high-risk factors associated with the survival

In this study, the relationship between 54 factors and were included in the univariate logistic regression analysis. The results showed that Age, Temperature, Heart rate, Pulse Oximetry, Lymphocyte, C- reactive protein, SAA, NLR, Tn-T, Pro–BNP, D–DIMER, ALT, ALB, GLU, HLN, Ca, LDH, PCT, IL-6, Pleural effusion and Heart enlargement were associated with poor prognosis in patients with severe Omicron pneumonia (*P* < 0.05). These factors were subsequently included in a multivariate logistic regression analysis (Table 4), which showed that Pulse Oximetry (*p* = 0.034, OR = 0.25, 95% CI: 0.07–0.9), LDH (*p* = 0.047, OR = 4.39, 95% CI: 1.02–18.93), SAA (*p* = 0.020, OR = 25.01, 95% CI:1.65–378.4), GLU (*p* = 0.022, OR = 5.49, 95% CI: 1.28–23.44), Ca (*p* = 0.029, OR = 0.23, 95% CI: 0.06–0.86), Pleural effusion (*p* = 0.008, OR = 8.04, 95% CI:1.73–37.46), and Heart enlargement (*p* = 0.008, OR = 8.94, 95% CI: 1.79–44.59) were independent prognostic factors affecting survival in patients with severe Omicron pneumonia. Multicollinearity among the variables included in the multivariate model was assessed using the variance inflation factor (VIF), and no significant multicollinearity was found (all VIF < 5). Patients with a combination of decreased pulse oximetry and Ca, elevated LDH, SAA, and GLU levels at baseline, as well as pleural effusion and heart enlargement tended to have a poorer clinical prognosis.

**TABLE 4:** Univariate and Multivariate analysis of potential prognostic factors identified in the training cohort.

Characteristic no. (%)	Univariate analysis		Multivariate analysis	
	OR (95% CI)	*P*-value	OR (95% CI)	*P*-value
Age, [median (IQR)]	2.589 (1.153–5.812)	0.021		
Male	0.847 (0.332–2.16)	0.728		
Vaccination	1.152 (0.53–2.504)	0.72		
**Symptoms**				
Cough	1.702 (0.777–3.732)	0.184		
Fever	1.771 (0.737–4.256)	0.202		
Breath shortness	1.327 (0.579–3.039)	0.503		
**Comorbidities**				
Hypertension	1.119 (0.515–2.433)	0.776		
Diabetes	1.714 (0.73–4.027)	0.216		
Cardiovascular disease	1.779 (0.806–3.924)	0.154		
Chronic lung disease	1.167 (0.399–3.415)	0.778		
Chronic liver disease	0.476 (0.048–4.737)	0.527		
Chronic kidney disease	1.053 (0.311–3.563)	0.934		
Cancer	1.571 (0.54–4.571)	0.407		
Autoimmune system disease	0.351 (0.038–3.255)	0.357		
**Basic vital signs (>cut-off)**				
Temperature	3.683 (1.394–9.728)	0.009		
Heart rate	2.875 (1.278–6.47)	0.011		
Breathing rate	2.142 (0.944–4.862)	0.068		
Pulse Oximetry	0.227 (0.096–0.54)	0.001	0.25 (0.07–0.9)	0.034
**Laboratory findings to admission (>cut-off)**				
WBC	2.062 (0.771–5.515)	0.149		
Neutrophil	2.363 (0.861–6.486)	0.095		
Lymphocyte	0.245 (0.093–0.645)	0.004		
C- reactive protein	4.49 (1.449–13.911)	0.009		
SAA	10.054 (1.164–86.815)	0.036	25.01 (1.65–378.4)	0.020
NLR	2.935 (1.314–6.554)	0.009		
Tn-T	2.957 (1.134–7.711)	0.027		
Pro–BNP	2.489 (1.117–5.547)	0.026		
D-DIMER	2.742 (1.231–6.106)	0.014		
AST	1.968 (0.869–4.46)	0.105		
ALT	0.277 (0.107–0.717)	0.008		
ALB	0.076 (0.01–0.601)	0.015		
GLU	6.851 (2.181–21.524)	0.001	5.49 (1.28–23.44)	0.022
HLN	3.091 (1.266–7.547)	0.013		
LDH	2.475 (1.041–5.883)	0.04	4.39 (1.02–18.93)	0.047
PCT	2.64 (1.112–6.267)	0.028		
IL-6	5.7 (1.98–16.412)	0.001		
Ca	0.387 (0.171–0.876)	0.023	0.23 (0.06–0.86)	0.029
K	0.708 (0.322–1.553)	0.389		
Na	0.521 (0.237–1.142)	0.104		
Cl	0.426 (0.169–1.076)	0.071		
**Imaging characteristics**				
**Nature of lesion**				
Grinding glass shadow mainly	0.793 (0.412–1.526)	0.487		
Solidity-based	1.284 (0.665–2.479)	0.457		
Bronchial wall thickening	1.122 (0.845–1.489)	0.426		
Thickening of blood vessels	0.698 (0.267–1.825)	0.464		
Tree bud levy	0.72 (0.126–4.114)	0.711		
Thickened lobular septa	2.1 (0.211–20.889)	0.527		
Intralobular septal thickening	2.1 (0.211–20.889)	0.527		
Stretch bronchiectasis	3.621 (0.408–32.14)	0.248		
Pleural effusion	2.76 (1.055–7.215)	0.038	8.04 (1.73–37.46)	0.008
Heart enlargement	4 (1.451–11.031)	0.007	8.94 (1.79–44.59)	0.008
Pericardial effusion	1.517 (0.628–3.661)	0.354		
Widening of the pulmonary artery	1.528 (0.699–3.339)	0.288		

### Construction of the nomogram

**FIGURE 5:**
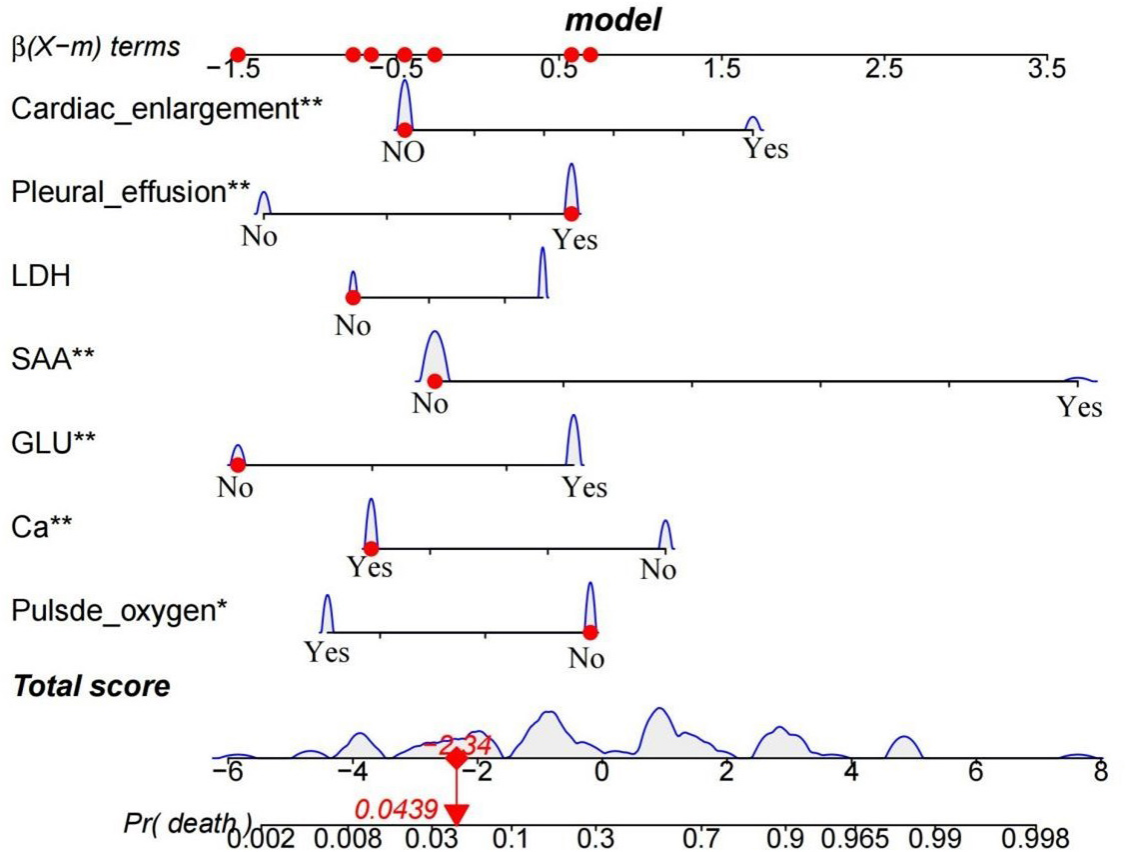
The final nomogram consisting of Pulse Oximetry, LDH, SAA, GLU, Ca, Pleural effusion and Heart enlargement is displayed. The graph shows a patient with a presumed combined pleural effusion, admitted with LDH 312.93, SAA 1134.5, GLU 7.5, Ca 2.05, and admission pulse oxygen 93% (indicated by red dots). The total score for this patient was calculated from the nomogram as 2.34, representing approximately 0.0439 of the probability of in-hospital death (indicated in the nomogram). Variables marked with * or ** are the primary factors affecting the total score calculation, with ** indicating a greater contribution.

In the training cohort, 63 (59.5%) patients were eventually cured and discharged, with an average length of hospital stay of 20.9 days; 43 (40.5%) patients experienced death during treatment, with an average length of hospital stay of 18.3 days. A nomogram model was developed to predict the risk of in-hospital death during treatment of patients with severe COVID-19 pneumonia. The final model included independent prognostic influences that were significant after multivariate logistic regression analysis: Pulse Oximetry, LDH, SAA, GLU, Ca, Pleural effusion, and Heart enlargement (Figure 5). The predicted risk of in-hospital death during treatment was determined by incorporating patient information into a risk scale to produce an overall score.

### Validation of the nomogram

**FIGURE 6:**
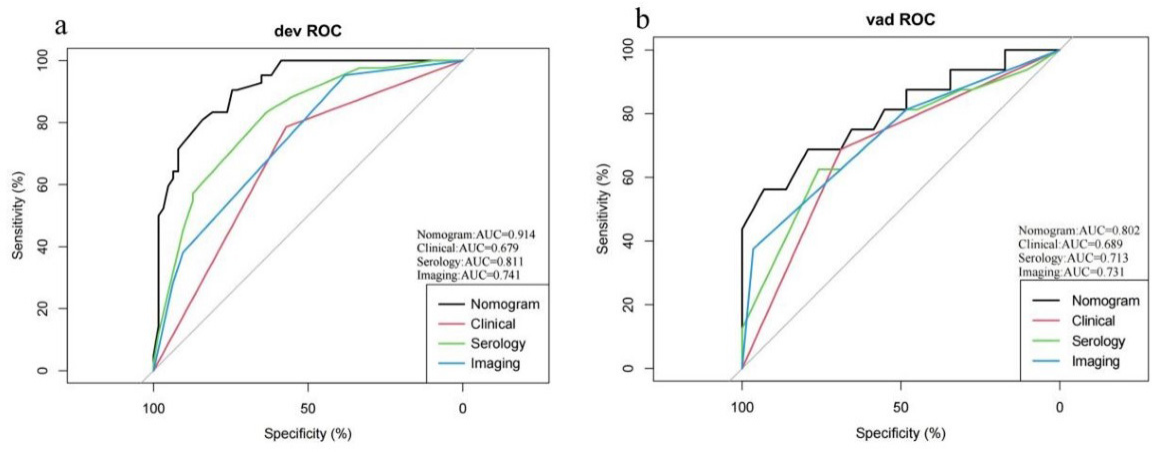
ROC curve and area under the subject’s working curve (AUC) of the nomogram in the training and validation cohort. **(a)** Indicate the ROC curve and AUC of the nomogram in predicting survival in the training cohort. **(b)** Indicate the ROC curve and AUC of the nomogram in predicting survival in the validation cohort.

**FIGURE 7:**
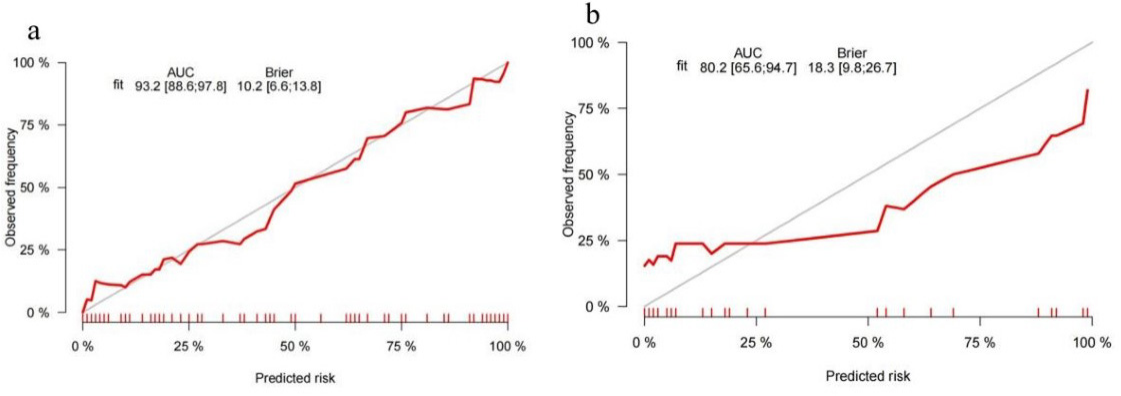
The calibration plot of the nomogram in the training and validation cohort. The calibration plot for predicting survival in the training cohort **(a)** and validation cohort **(b)**. Actual rate of survival is shown on the y-axis, and the nomogram- predicted probability of survival is shown on the x-axis.

In the validation cohort, 29 patients were eventually discharged with a cure, with a mean length of hospital stay of 21.7 days; 16 patients experienced death during treatment, with a mean length of hospital stay of 17.7 days. The AUC for the training cohort was 0.914 and for the validation cohort was 0.802 (Figure 6). A balance test indicated that most baseline characteristics were comparable between the training and validation cohorts, although some differences were observed (e.g., gender distribution, prevalence of hypertension). This is a common challenge in single-center studies with limited sample size and random split, and it may partially account for the observed drop in the validation AUC. We also compared the performance of our nomograms with the performance of single factor categories for prediction, such as baseline clinical characteristics (Pulse Oximetry), serology (LDH, SAA, GLU, and Ca) and imaging (Pleural effusion and Heart enlargement). The results show that combining multiple types of baseline features nomograms is more advantageous than using a single factor to predict patient prognosis (Figure 6). We also carried out the plotting of calibration curves for the training and validation cohort survival prediction (Figure 7). This was used to illustrate the agreement between the nomogram’s predictions of patient survival prognosis and actual observations. The results of the study show that the model has good overall predictive efficacy. The predictive performance of the full nomogram was significantly superior to that of the single strongest predictor, Pulse Oximetry, in the training cohort (DeLong test, *p* < 0.05).

### Clinical application of the nomogram

**FIGURE 8:**
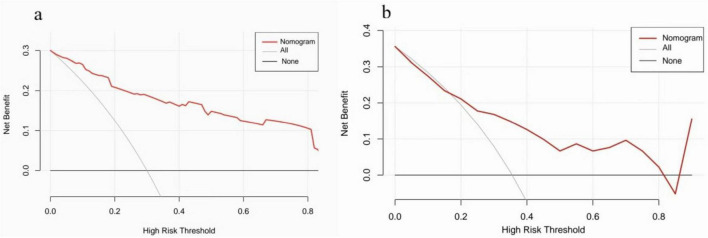
Decision Curve Analysis (DCA) of the nomogram. The DCA assesses the clinical value of the nomogram (red line) by quantifying its net benefit compared to the “Treat All” (gray line) and “Treat None” (black line) strategies over various threshold probabilities. The shaded area highlights the range (approx. 10%–65%) where the nomogram provides a clear clinical net benefit. The consistent performance in both the training **(a)** and validation **(b)** cohorts underscores the robustness of the model for potential clinical application.

An evaluation of the clinical applicability of the risk prediction nomogram was conducted using DCA grounded in net benefit and threshold probabilities. The DCA results indicated that our risk prediction nomogram provided a greater net benefit across a broad spectrum of threshold probabilities in both the training and validation cohorts (Figure 8). The DCA indicated that the nomogram provided a positive net benefit for threshold probabilities between approximately 10% and 65%. The consistent performance in both the training (a) and validation (b) cohorts underscores the robustness of the model for potential clinical application.

## Discussion

The COVID-19 pandemic has lasted nearly 3 years and probably will continue to be a global concern (21). The increasing prevalence of the COVID-19 pandemic has attracted the attention of the entire world since China’s epidemic prevention and control policies were modified in December 2022 (22). During this period, the Chinese Centre for Disease Control and Prevention conducted continuous surveillance of the epidemic, and from 26 September 2022 to 23 January 2023, a total of 18,906 cases were reported nationwide, with valid sequences of the genomes of indigenous cases of the COVID-19, all of which were Omicron mutants (22). Omicron was first identified in South Africa and designated as a VOC by WHO; it had an unprecedented number of mutations and increased transmissibility (23). Despite studies indicating that it may be of lower clinical severity than Delta (24, 25), it has also been found to have a faster rate of spread and higher immune escape than Delta (23, 26). The Omicron has rapidly spread throughout the world and has replaced the Delta as the dominant strain, causing countries to experience multiple outbreak peaks (21). In this study, the rate of severe infection among COVID-19 patients admitted to Shandong Provincial Hospital was 8.70%, and the death rate of severe patients was 39.0%. Following the outbreak of COVID-19 in China, a country with a large population and a large older adult population, the health care system is under extreme strain. Patients at high risk for a poor prognosis can be treated with active supportive care if their prognosis is improved by early prognostic prediction and risk stratification. The significance of accurate and effective prognostic models for COVID-19 clinical management.

In previous studies, various clinical information such as baseline age (*P* = 0.014), CRP (*P* = 0.035) (27), AST (*P* = 0.027), and D-Bil (*P* = 0.001) (28) levels may be significantly associated with disease regression in patients with COVID-19. We developed a nomogram for the risk of death associated with severe (Omicron) COVID-19 pneumonia based on clinical symptoms, serological indicators, and imaging characteristics and validated the nomogram using an independent internal validation cohort, which confirmed the good predictive efficacy of the nomogram.

In this study, we identified a combination of Pleural-effusion (PE) and Cardiac-enlargement in 69.5% and 20.5% of severe (Omicron) COVID-19 pneumonia, respectively, and this imaging characteristic was significantly associated with an increased risk of patient mortality. Several previous studies found a substantial association between the occurrence of PE and the severity of the disease (OR 3.31, 95% CI [2.03–5.38]) (29). It could be that PE is generally associated with more severe interstitial involvement in COVID-19, and severe PE frequently results in impaired gas exchange, prolonging the patient’s hospital stay and even becoming a risk factor for mortality (30). At the same time, Cardiac enlargement is usually associated with the occurrence of combined hypertension, chronic cardiac insufficiency, myocardial injury and viral myocarditis, while previous studies have found that the occurrence of chronic cardiac insufficiency (HR, 4.28; 95% CI, 1.14–16.13) (31) and myocardial injury (*P* < 0.05) (32) are significantly associated with an increased risk of death from disease in patients. Heart enlargement may be caused by a combination of chronic cardiac insufficiency and myocardial injury, an imbalance between the increased metabolic demand induced by infection and the reduced cardiac reserve, resulting in heart failure and thus influencing the patient’s clinical prognosis. The presence of pleural effusion in COVID-19 may be related to increased pulmonary capillary permeability, systemic inflammation, or concomitant cardiac failure. Cardiac enlargement often reflects pre-existing or acute stress-induced cardiopathy. In the context of Omicron, which has a different tropism compared to earlier variants, these findings might signify a significant cardiopulmonary burden. In order to actively enhance the prognosis of patients with COVID-19 who have pleural effusion and cardiac enlargement, we should be vigilant in the early stages. In addition, in several previous studies, we found that patients’ combined Hypoxemia on admission (*P* = 0.007) (33), liver and kidney impairment (Creatinine: *P* = 0.02; Blood urea nitrogen: *P* < 0.001) (34) and elevated blood glucose (OR: 7.629, 95% CI: 1.391–37.984) (35) were associated with an increased risk of death in patients. In this study, we likewise found that elevated baseline combined HLN, GLU with reduced Ca^2+^ levels and Pulsde-oxyge were significantly associated with the prognosis of COVID-19 patients with severe omicron infection. This is in concordance with the results of previous studies. In this study, we identified a combination of Pleural-effusion (PE) and Cardiac-enlargement in 69.5% and 20.5% of severe (Omicron) COVID-19 pneumonia, respectively, and this imaging characteristic was significantly associated with an increased risk of patient mortality. Several previous studies found a substantial association between the occurrence of PE and the severity of the disease (OR 3.31, 95% CI [2.03–5.38]) (29) It could be that PE is generally associated with more severe interstitial involvement in COVID-19, and severe PE frequently results in impaired gas exchange, prolonging the patient’s hospital stay and even becoming a risk factor for mortality (30). At the same time, Cardiac enlargement is usually associated with the occurrence of combined hypertension, chronic cardiac insufficiency, myocardial injury and viral myocarditis, while previous studies have found that the occurrence of chronic cardiac insufficiency (HR, 4.28; 95% CI, 1.14–16.13) (31) and myocardial injury (*P* < 0.05) (32) are significantly associated with an increased risk of death from disease in patients. Heart enlargement may be caused by a combination of chronic cardiac insufficiency and myocardial injury, an imbalance between the increased metabolic demand induced by infection and the reduced cardiac reserve, resulting in heart failure and thus influencing the patient’s clinical prognosis. In order to actively enhance the prognosis of patients with COVID-19 who have pleural effusion and cardiac enlargement, we should be vigilant in the early stages. In addition, in several previous studies, we found that patients’ combined Hypoxemia on admission (*P* = 0.007) (33), liver and kidney impairment (Creatinine: *P* = 0.02; Blood urea nitrogen: *P* < 0.001) (34) and elevated blood glucose (OR: 7.629, 95% CI: 1.391–37.984) (35) were associated with an increased risk of death in patients. In this study, we likewise found that elevated baseline combined HLN, GLU with reduced Ca^2+^ levels and Pulsde-oxyge were significantly associated with the prognosis of COVID-19 patients with severe omicron infection. The wide confidence intervals for some predictors in the multivariate model (e.g., SAA) likely reflect the limited sample size of severe cases and the low frequency of certain high-risk characteristics, leading to less precise estimates. This is in concordance with the results of previous studies. The wide confidence intervals for some predictors in the multivariate model (e.g., SAA) likely reflect the limited sample size of severe cases and the low frequency of certain high-risk characteristics, leading to less precise estimates. Furthermore, some risk factors identified, such as elevated glucose levels, are modifiable. Although our study cannot establish causality due to its observational design, future research could employ methods like target trial emulation to explore causal effects of interventions on these factors (36).

Furthermore, some risk factors identified, such as elevated glucose levels, are modifiable. Although our study cannot establish causality due to its observational design, future research could employ methods like target trial emulation to explore causal effects of interventions on these factors (36).

Owing to China’s sustained policy of dynamic clearance for outbreak prevention and control, China experienced only a transient outbreak of COVID-19 at the outbreak’s onset in 2019, and its incidence has remained low in China since then. The majority of clinical prognostic models for Chinese COVID-19 patients were developed based on clinical information available at the time, whereas the current worldwide widespread prevalence of the omicron strain differs from the Delta strain at the outbreak’s onset in terms of virulence, pathogenic characteristics, and disease regression. Consequently, clinical prediction models based on prevalence data of Delta strains have limitations in their clinical application at the present time. Due to differences in ethnicity, dietary practices, and climate, it is uncertain whether clinical prognostic models based on the omicron strain in other countries can be applied to patients with COVID-19 in China. As a result, we developed a mortality risk prediction model using baseline data from Chinese patients infected with the omicron strain of COVID-19.

Firstly, the nomogram combined a variety of clinical information, such as basic information, laboratory tests, and imaging examinations, to provide a higher accuracy of prediction than a single type of factor. Secondly, the nomogram combines for the first time two imaging data, pleural effusion and cardiac enlargement, for prediction, showing good clinical predictive efficacy, which could be followed by a more in-depth exploration of its clinical causes affecting prognosis. Finally, to our knowledge, the nomogram is the first prognostic risk assessment model based on critically ill COVID-19 patients infected with the Chinese omicron strain.

Undoubtedly, this study has unavoidable limitations. This is a retrospective, single-center study that may be susceptible to unavoidable bias. The outcomes of hospitalized patients may vary based on their medical condition, regional distribution, care, and infection count. Another limitation of our study is the unavailability of the original analysis dataset, which precluded the calculation of diagnostic accuracy metrics for individual cut-off values and a direct comparison with established clinical scores like CURB-65. Furthermore, we did not compare the performance of our nomogram with existing prognostic scores such as CURB-65 or the 4C Mortality Score. Future studies should include such comparisons to better establish the incremental value of the combined clinical and imaging features identified here. Due to the retrospective nature of our study, we were unable to establish a correlation between patient prognosis and viral load. This research is based on the period of hospital admission, not the time between symptom onset and hospital discharge. It is difficult to obtain the necessary information if a patient does not go to the hospital immediately after discovering symptoms. Despite these limitations, we were able to construct a highly accurate model for predicting COVID-19 patients’ in-hospital survival. This prediction model is intended to facilitate the clinical application of COVID-19 management and enhance patient prognoses. However, the sample size of severe COVID-19 patients is relatively small, and the event per variable ratio in the multivariate model is below the recommended threshold, which may lead to overfitting. The drop in AUC from the training to validation cohort suggests some overfitting, and the model requires further validation in larger cohorts. Moreover, our validation was internal, and the model requires external validation in independent cohorts from different regions to confirm its generalizability.

In conclusion, we identified a combination of Pleural-effusion (PE) and Cardiac-enlargement in 69.5% and 20.5% of severe (Omicron) COVID-19 pneumonia, respectively, and this imaging characteristic was significantly associated with an increased risk of patient mortality. In this study, we likewise found that elevated baseline combined HLN, GLU with reduced Ca^2+^ levels and Pulsde-oxyge were significantly associated with the prognosis of COVID-19 patients with severe omicron infection. Therefore, based on these findings, we preliminary developed a mortality risk prediction model using baseline data from Chinese patients infected with the COVID-19, which showed good clinical prediction and can be followed up with a more in-depth exploration of the clinical reasons why it affects prognosis. It is important to note that the population with severe COVID-19 is heterogeneous, and our model may not perform equally across all subgroups. Future studies should explore stratified analyses based on factors such as age, comorbidities, or vaccination status to refine the prediction model (37). Regarding clinical application, this nomogram could be integrated into the emergency department or admission workflow for patients diagnosed with severe Omicron pneumonia. By quickly inputting the seven readily available parameters (SpO2, LDH, SAA, GLU, Ca, Pleural Effusion, Heart Enlargement), clinicians could obtain an individual patient’s mortality risk score. This could aid in early triage, guiding the intensity of monitoring (e.g., direct admission to ICU for high-risk scores), and informing discussions with patients and families about the prognosis. Future work should focus on developing a digital version of this nomogram and defining specific risk thresholds for clinical decision-making.

## Data Availability

The raw data supporting the conclusions of this article will be made available by the authors, without undue reservation.
